# Remarkable Case of Ascites

**Published:** 1816-06

**Authors:** Charles Seaman Gaye

**Affiliations:** Member of the College of Surgeons.


*For the London Medical and Physical Journal.*

*Remarkable Case of Ascites;* by CHARLES SEAMAN GAYE, Esq. Member of the College of Surgeons.

NEVER having met with a case recorded in which the fluid extracted, at different times, from the cavity of the abdomen, in a *living* subject, *averaged* so prodigious a quantity as in the following ascitical patient, which has recently occurred in my practice, I have been induced to obtrude myself on your attention, by requesting its insertion in the Medical Journal; should you, upon its perusal, deem it worthy of the public notice, and a place in your highly useful and valuable publication.

Elizabeth Gee, of Silsoe, aged forty-five, married fourteen years, had catamenia regularly to the usual period of their termination, notwithstanding the following hydropic disease had existed for several years. She experienced two abortions during the conjugal state, but never went to the full period of uterine gestation, having laboured under ascitical symptoms for nearly eight years, and the usual hydragogue and diuretic remedies having been repeatedly administered under various eminent professional gentlemen, without the least benefit; but still gradually increasing in her enormous dimensions, and extreme sufferings. She was finally prevailed upon to submit to the operation of paracentesis abdominis, which I performed on her on Saturday the 29th day of May, 1813. From the surprising distension of the abdominal parietes, very little impediment was experienced to the introduction of the triangular-pointed trocar, nor was there any expression of pain from the patient. The spot chosen for its insertion was about centrical, between the umbilicus and the anterior superior spinous process of the left ilium. The whole fluid ran freely off in about thirty minutes; slight interruptions, however, were occasionally and purposely given by the finger placed over the mouth of the canula, to guard against the probable occurrence of syncope, by a too hasty discharge; which, by the assistance of cordials also, and a constant uniform and powerful compression by appropriate bandages over the abdomen, was prevented; and she was carried to bed amazingly relieved and cheerful. The fluid was received into four large wooden pails and an earthen pan of similar dimensions, and, upon being carefully measured, amounted to *fifty-two standard beer quarts, one pint*, of a semi-transparent greenish appearance, inodorous, and of a slightly-gelatinous consistence, weighing 120 pounds avoirdupois. On the Tuesday following she walked down stairs, and from that period progressively recovered her strength, and was, about the 20th of June, in better health and spirits than what she had experienced for several preceding years, being capable of walking a mile or two without inconvenience.

The fluid having again accumulated so as to render her situation insupportable, she became very desirous again for the operation; which she submitted to on the 28th day of February, 1814; when there were drawn off *forty-nine beer quarts, one pint,* of fluid of a similar colour and consistence.

On the 12th day of April, 1815, she measured round the

 body, body, over the umbilicus (which was enlarged to the size of a pigeon's egg), *sixty-one inches*, on which day I again took from her *forty-three quarts.*

About the latter end of March, she again became, in consequence of her sufferings, very impatient for the operation; and, on the 6th day of April, 1816, at four o'clock in the afternoon, I again extracted *thirty-five beer quarts* of fluid (standard measure), in the presence of a physician and surgeon, &c. She bore the operation as usual, with great fortitude and composure; was placed in bed at half-past four, in an apparently similar state to what she had at other times experienced after former operations; talked low, but rather cheerful; and at eleven o'clock at night desired the attendants to go to bed: a change, however, soon afterwards took place, and she expired about twelve o'clock the same night. Not the slightest appearance of blood was discovered during or succeeding either of the operations; and the wounds healed favourably.

*Shefford, Bedfordshire;*

